# Supplementary material for: Genomics of Re-Emergent Aeromonas salmonicida in Atlantic Salmon Outbreaks
Source: Microorganisms. 2023 Dec 29;12(1):64. doi: 10.3390/microorganisms12010064 (PMC10819690; doi:10.3390/microorganisms12010064)
Supplement: Supplementary file 1 [file microorganisms-12-00064-s001.zip › Supplementary figures.pdf]

Pairwise average nucleotide identities

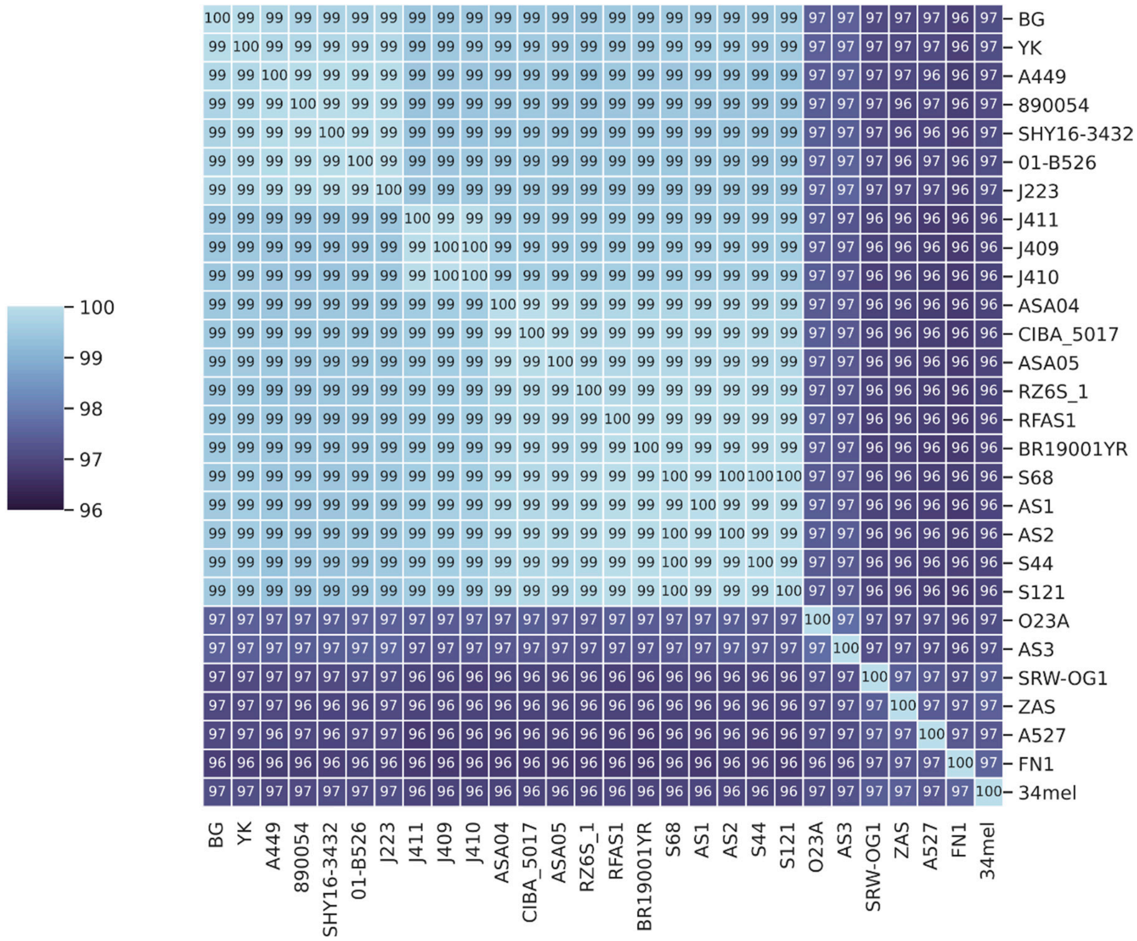

Supplementary Figure S1. Pairwise average nucleotide identity (ANI) values among *Aeromonas salmonicida* strains. ANI values were computed with the FastANI tool.

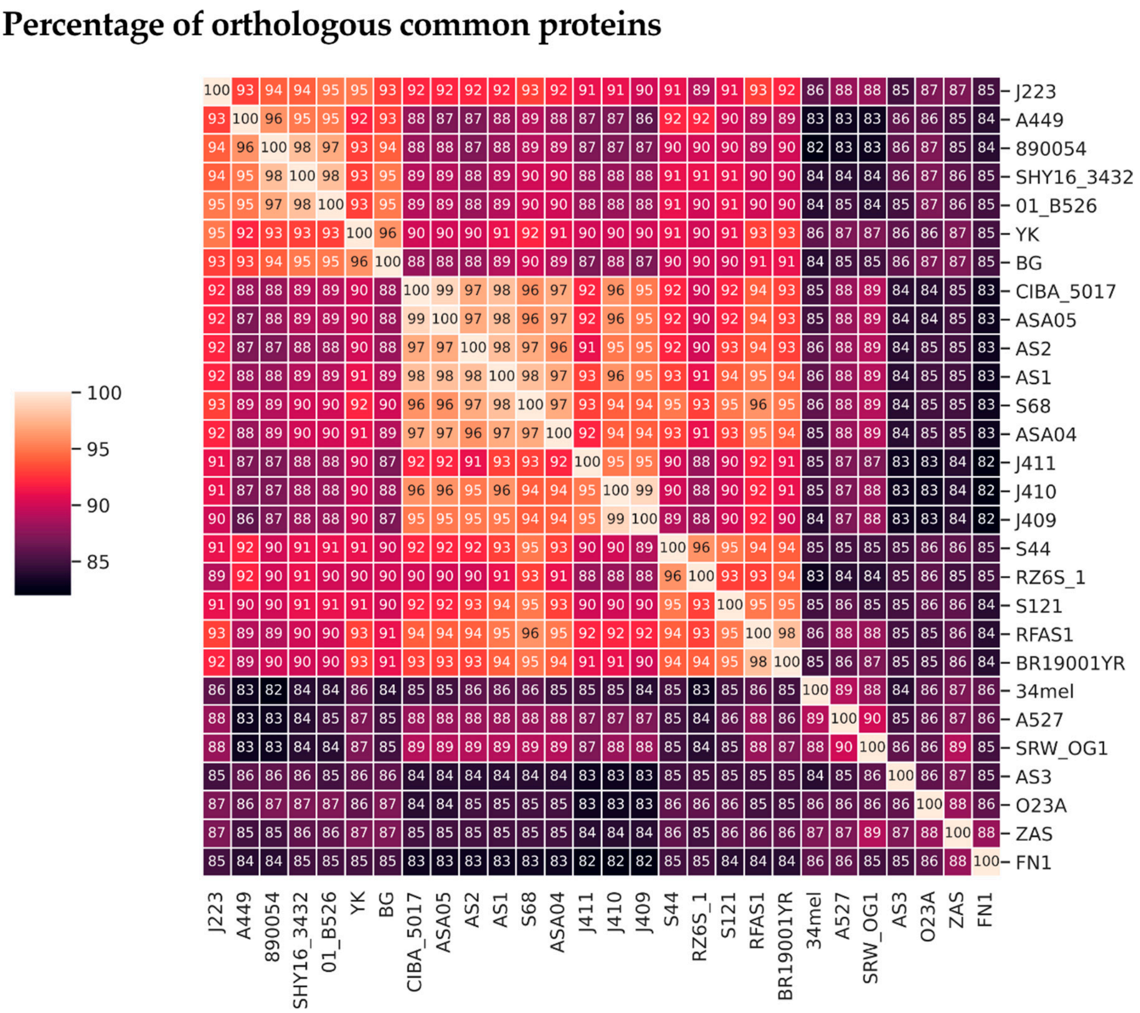

Supplementary Figure S2. Percentage of orthologous common proteins among *Aeromonas salmonicida* strains. Gene orthologous families as predicted by the PIRATE tool with default aminoacidic similarity threshold were counted through pairwise comparison among *A. salmonicida* strains. Percentage calculated with respect to the total number of gene families of the strain where this number is greater, for each pairwise comparison.

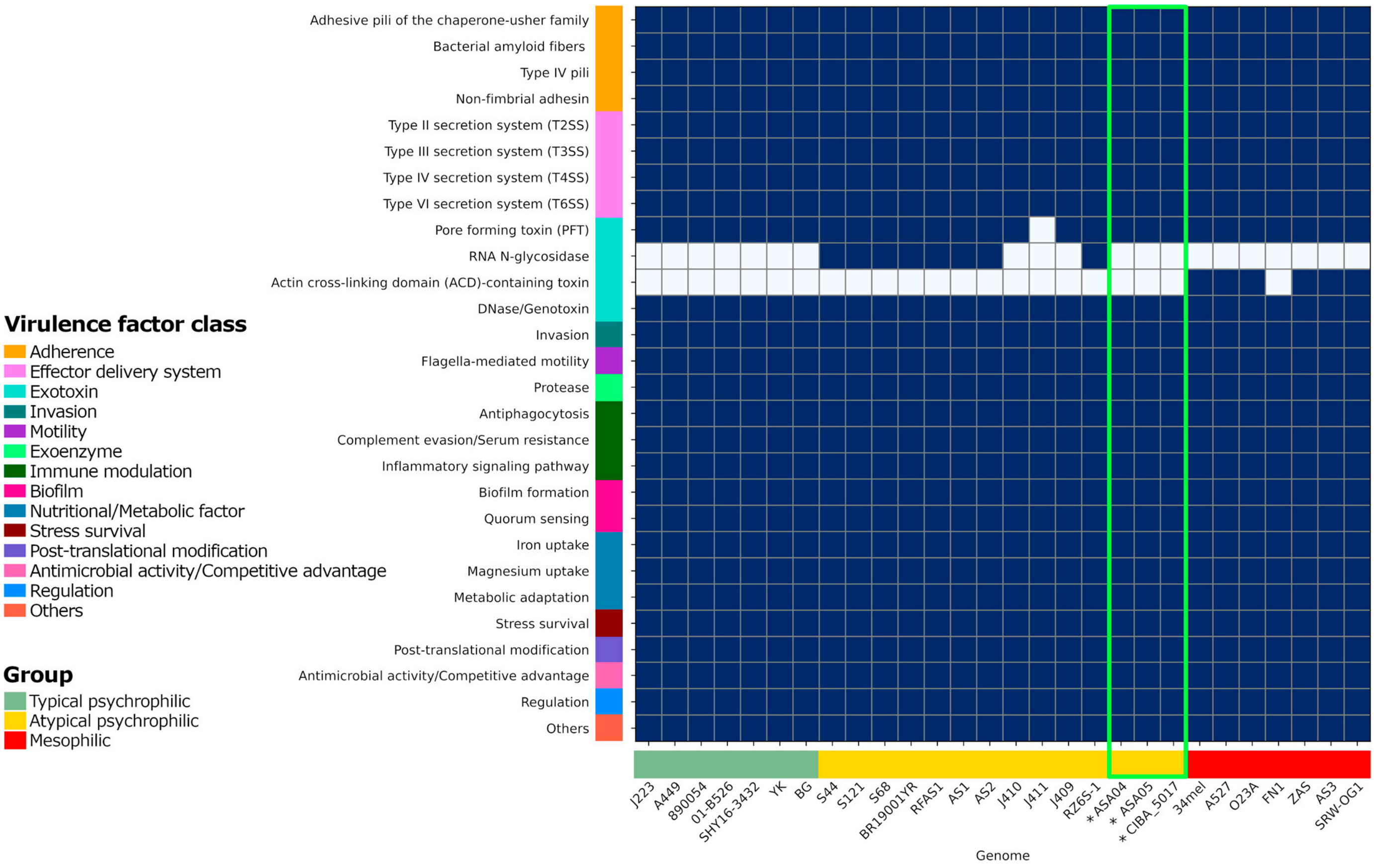

Supplementary Figure S3. Virulence factors landscape of *Aeromonas salmonicida* complete genomes. The presence-absence of the factors and their subclasses were predicted based on local alignments with the VFDB entries. The presence or the absence of the factor subclass are represented in blue and white, respectively. *: Genomes reported in this study.
